# Comparative Evaluation of Salivary Chemerin and Matrix Metalloproteinase-9 Levels in Healthy Individuals, Patients With Periodontitis, and Patients With Oral Squamous Cell Carcinoma: A Cross-Sectional Study

**DOI:** 10.7759/cureus.110757

**Published:** 2026-06-12

**Authors:** Prasad Shirke, Siddhartha Varma, Girish Suragimath, Sameer A Zope, Vaishali N Mashalkar, Apurva V Kale, Anand Gudur

**Affiliations:** 1 Department of Periodontology, School of Dental Sciences, Krishna Vishwa Vidyapeeth (Deemed to be University), Karad, IND; 2 Department of Oncology, Krishna Vishwa Vidyapeeth (Deemed to be University), Karad, IND

**Keywords:** chemerin, mmp-9, oral squamous cell carcinoma, periodontitis, salivary biomarker

## Abstract

Background: Periodontitis and oral squamous cell carcinoma (OSCC) are chronic inflammatory conditions linked to systemic health and share biological pathways involving immune dysregulation, oxidative stress, and tissue destruction.

Aim: The aim of the study is to evaluate and compare salivary chemerin and matrix metalloproteinase-9 (MMP-9) levels among healthy individuals, periodontitis patients, and OSCC patients, and to assess their potential diagnostic utility.

Materials and methods: A cross-sectional study was conducted on 90 participants divided into three groups (n = 30 each): healthy controls, periodontitis patients, and OSCC patients. Unstimulated saliva samples were obtained and analyzed for chemerin and MMP-9 using enzyme-linked immunosorbent assay (ELISA). Periodontal parameters, including oral hygiene index-simplified (OHI-S), Russell’s index, probing pocket depth (PPD), and clinical attachment loss (CAL), were recorded. Analysis of variance (ANOVA), Tukey’s post hoc test, and Pearson correlation were used for statistical analysis.

Results: Salivary chemerin and MMP-9 levels were significantly elevated in both periodontitis and OSCC patients compared to healthy individuals, with the highest levels observed in the OSCC group. MMP-9 demonstrated a stronger association with periodontal tissue destruction. Analysis revealed a direct association between chemerin and MMP-9 concentrations.

Conclusion: Salivary chemerin and MMP-9 levels were significantly higher in periodontitis and OSCC patients than in healthy individuals, with the highest levels observed in the OSCC group. Elevated biomarker levels were associated with worsening periodontal parameters, indicating their role in inflammation, tissue destruction, and tumor progression. These findings suggest a possible inflammatory link between periodontitis and OSCC and support the potential of salivary chemerin and MMP-9 as non-invasive biomarkers for disease diagnosis and monitoring.

## Introduction

The periodontium, which includes the gingiva, periodontal ligament, cementum, and alveolar bone, plays an essential role in maintaining oral health and supporting the teeth [1]. Periodontitis is a chronic inflammatory disease caused by the interaction between pathogenic microorganisms and the host immune response, resulting in progressive destruction of periodontal tissues [2]. Recent evidence suggests that chronic periodontal inflammation may also be associated with oral squamous cell carcinoma (OSCC), as both conditions involve inflammatory and immune-mediated mechanisms [3].

This inflammatory environment, rich in cytokines and oxidative stress, may also contribute to deoxyribonucleic acid (DNA) damage and altered cellular activity [3,4]. Several studies suggest that periodontal disease is linked with an increased risk of systemic conditions, including various cancers [4,5].

OSCC is the most prevalent oral malignancy and remains a major health concern, especially in populations with high tobacco and alcohol use [6]. Delayed diagnosis is common because early symptoms are absent. Chronic inflammation plays a key role in its development by promoting genetic changes, tumor growth, and metastasis [7].

Shared biological mechanisms, including microbial dysbiosis, persistent inflammation, and oxidative stress, help explain the link between periodontitis and OSCC [8]. These processes can lead to cellular damage, DNA alterations, and tumor progression [9,10]. Saliva is a valuable diagnostic fluid due to its non-invasive collection and its ability to reflect both local and systemic conditions [11]. Chemerin is an inflammatory adipokine involved in immune cell recruitment and regulation. Matrix metalloproteinase-9 (MMP-9) is a matrix-degrading enzyme that contributes to connective tissue breakdown in periodontitis and facilitates tumor invasion and metastasis in OSCC [12,13].

The study aims to evaluate and compare salivary chemerin and MMP-9 levels among healthy individuals, patients with periodontitis, and patients with OSCC, and to assess their relationships with clinical periodontal parameters, oral hygiene index-simplified (OHI-S), Russell's index, probing pocket depth (PPD), and clinical attachment level (CAL), to determine the association between biomarker expression and disease severity.

The findings are expected to determine whether significant differences in biomarker levels exist among the study groups and to explore their association with periodontal status and OSCC. The study may provide further insight into the inflammatory mechanisms shared by periodontitis and OSCC and contribute to understanding the potential roles of salivary chemerin and MMP-9 as non-invasive surrogate biomarkers of inflammatory burden in both periodontitis and OSCC. The null hypothesis of the study was that salivary chemerin and MMP-9 levels would not differ significantly among healthy individuals, periodontitis patients, and OSCC patients, and would show no significant association with periodontal clinical parameters.

## Materials and methods

Study design

The present cross-sectional study was done at the Department of Periodontology, School of Dental Sciences, Karad, following approval from the Ethical Committee of Krishna Vishwa Vidyapeeth (Deemed to be University), Karad (Ethical number: 006/2024-2025).

Sample size selection

The sample size was calculated using G*Power software (Version 3.1, Heinrich Heine University, Düsseldorf, Germany). With a significance level (α) of 0.05 and a statistical power of 80%, and based on available literature, an effect size of 0.40 was considered. The minimum required sample size was estimated to be 84 participants. To compensate for potential dropouts and incomplete data, 90 subjects were recruited and evenly allocated to three groups (30 participants per group).

Inclusion criteria

Individuals who provided informed consent and had a clinical diagnosis of periodontitis, as defined by the 2017 Classification of Periodontal and Peri-implant Diseases and Conditions [14], were included in the study. Those diagnosed with OSCC through clinical examination and confirmed by a histopathological report were identified and enrolled. Following a comprehensive clinical assessment, participants were assigned to the healthy, periodontitis, and OSCC groups.

Exclusion criteria

Individuals were excluded from the study if they had a history of systemic disorders, were pregnant or breastfeeding, had undergone periodontal treatment in the last three months, or were currently taking antibiotics, antiplatelet medications, or anti-inflammatory drugs. Additionally, those actively receiving treatment for OSCC, including chemotherapy, radiotherapy, or surgical intervention, were also excluded.

Clinical examination

**Figure 1:**
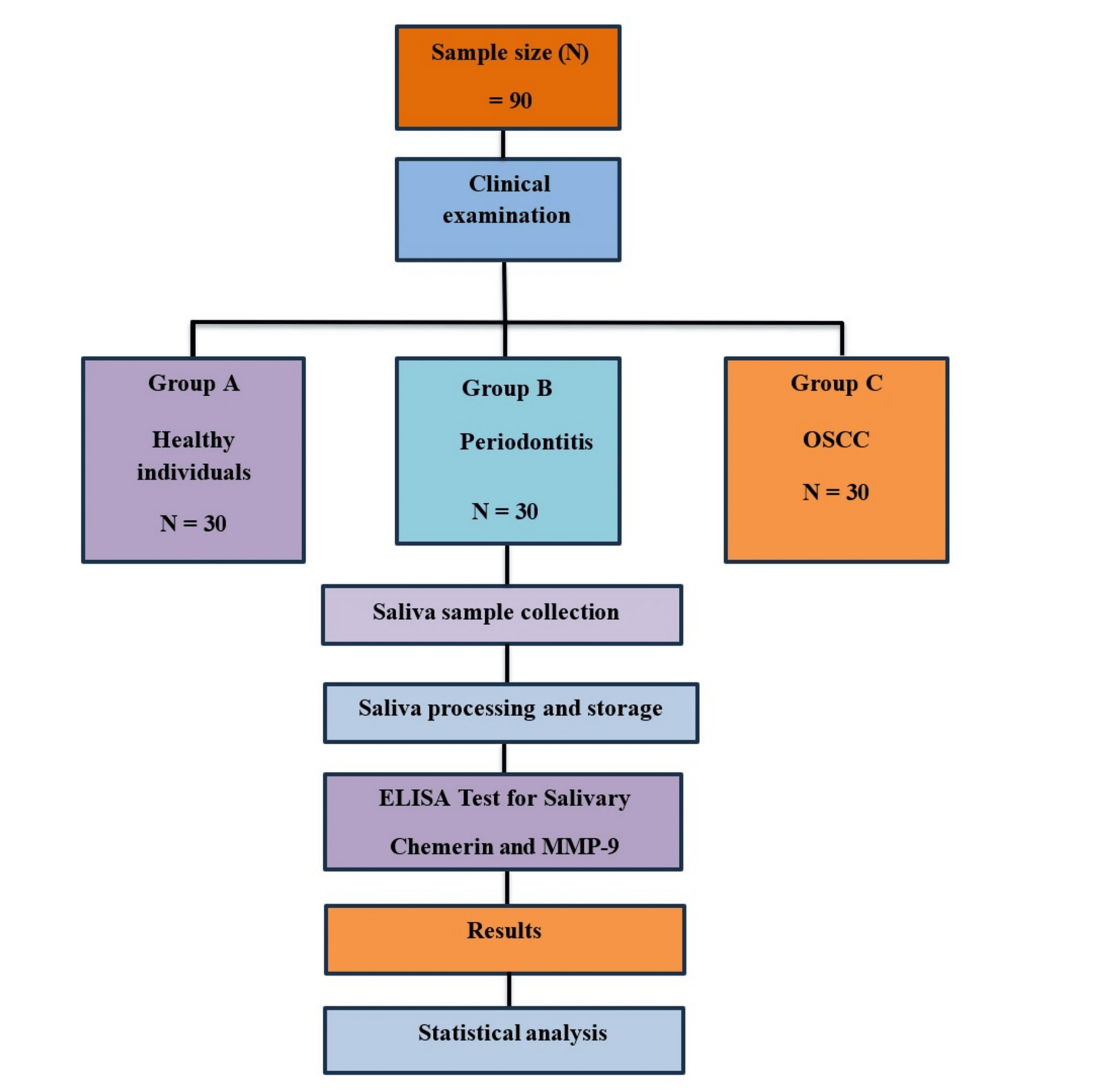
Overview of the study methodology. OSCC: oral squamous cell carcinoma; ELISA: enzyme-linked immunosorbent assay; MMP-9: matrix metalloproteinase-9

A total of 90 subjects underwent a detailed periodontal assessment. All periodontal clinical measurements were performed by a single calibrated and experienced periodontist to minimize measurement variability. Clinical evaluations included the OHI-S [15], Russell’s periodontal index [16], PPD for all teeth, and CAL measurement. Participants were allocated into three groups: Group A (n = 30) comprised healthy individuals; Group B (n = 30), patients with periodontitis; and Group C (n = 30), those diagnosed with OSCC. The study design is shown in Figure 1.

Saliva sample collection

An unstimulated whole saliva sample of 2 mL from all three groups was collected between 10:00 AM and 12:00 PM, using a modified technique based on Navazesh [17]. Participants were advised to refrain from eating, drinking, or brushing their teeth for at least one hour before the sample collection. To minimize contamination, clinical evaluations were conducted at least one hour before saliva sampling. Participants were first asked to swallow their saliva, then to allow the remaining saliva to accumulate passively over their lower lip for five minutes before transferring it to a sterile tube.

Saliva sample storage

After collection, the saliva was immediately centrifuged (Plasto Crafts Super Spin R V/FM, Plastocrafts Industries (P) LTD, Mumbai, India) for 15 minutes at 1,000 × g to remove particulate matter and cellular debris, and the clarified supernatant was promptly divided into aliquots and frozen at −80°C until assayed.

Biomarker analysis

The concentrations of salivary chemerin and MMP-9 were measured using enzyme-linked immunosorbent assay (ELISA) kits (ELK Biotechnology Co. Ltd., Wuhan, China), following the guidelines as per the manufacturer in the Microbiology laboratory of Krishna Vishwa Vidyapeeth (Deemed to be University), Karad.

Detection of Human Chemerin Levels in Saliva

Human CHEM (Chemerin) ELISA Kit (ELK Biotechnology Co. Ltd.) (Catalog Number ELK1953 96): sensitivity: 0.069 ng/mL; detection range: 0.16-10 ng/mL. The test principle applied in this kit is a sandwich enzyme immunoassay. The microtiter plate provided in this kit has been pre-coated with an antibody specific to CHEM. Standards or samples are added to the appropriate microtiter plate wells, then with a biotin-conjugated antibody specific to CHEM. Next, avidin conjugated to horseradish peroxidase (HRP) is added to each microplate well and incubated. After the TMB substrate solution is added, only those wells that contain CHEM, biotin-conjugated antibody, and enzyme-conjugated avidin will exhibit a change in color. The enzyme-substrate reaction is terminated by the addition of sulphuric acid solution, and the color change is measured spectrophotometrically at a wavelength of 450 nm ± 10 nm. The concentration of CHEM in the samples is then determined by comparing the optical density (OD) of the samples to the standard curve.

Detection of Human MMP-9 Levels in Saliva

Human MMP-9 ELISA Kit (ELK Biotechnology Co. Ltd.) (Catalog Number ELK1262): sensitivity: 0.059 ng/mL; detection range: 0.16-10 ng/mL. The test principle applied in this kit is a sandwich enzyme immunoassay. The microtiter plate provided in this kit has been pre-coated with an antibody specific to Human MMP-9. Standards or samples are added to the appropriate microtiter plate wells, then with a biotin-conjugated antibody specific to Human MMP-9. Next, avidin conjugated to HRP is added to each microplate well and incubated. After the TMB substrate solution is added, only those wells that contain Human MMP-9, biotin-conjugated antibody, and enzyme-conjugated avidin will exhibit a change in color. The enzyme-substrate reaction is terminated by the addition of sulphuric acid solution, and the color change is measured spectrophotometrically at a wavelength of 450 nm ± 10 nm. The concentration of Human MMP-9 in the samples is then determined by comparing the OD of the samples to the standard curve.

Statistical analysis

Clinical and salivary biomarker data were analyzed using IBM SPSS Statistics for Windows, version 21.0 (IBM Corp., Armonk, NY, USA). Intergroup comparisons were conducted with one-way analysis of variance (ANOVA), followed by Tukey’s post hoc test. Pearson’s correlation analysis assessed associations between periodontal parameters and salivary biomarkers. Statistical significance was set at p < 0.05.

## Results

This cross-sectional study evaluated and compared salivary levels of chemerin and MMP-9 in healthy individuals, patients with periodontitis, and those diagnosed with OSCC. Clinical periodontal parameters and salivary biomarker levels were analyzed and compared among the study groups.

In terms of gender distribution, the groups were comparable: Group A comprised 56% men and 44% women; both Groups B and C had 56% men and 44% women, and Group C had 60% men and 40% women. The difference in gender distribution was not statistically significant (p = 0.94).

**Figure 2:**
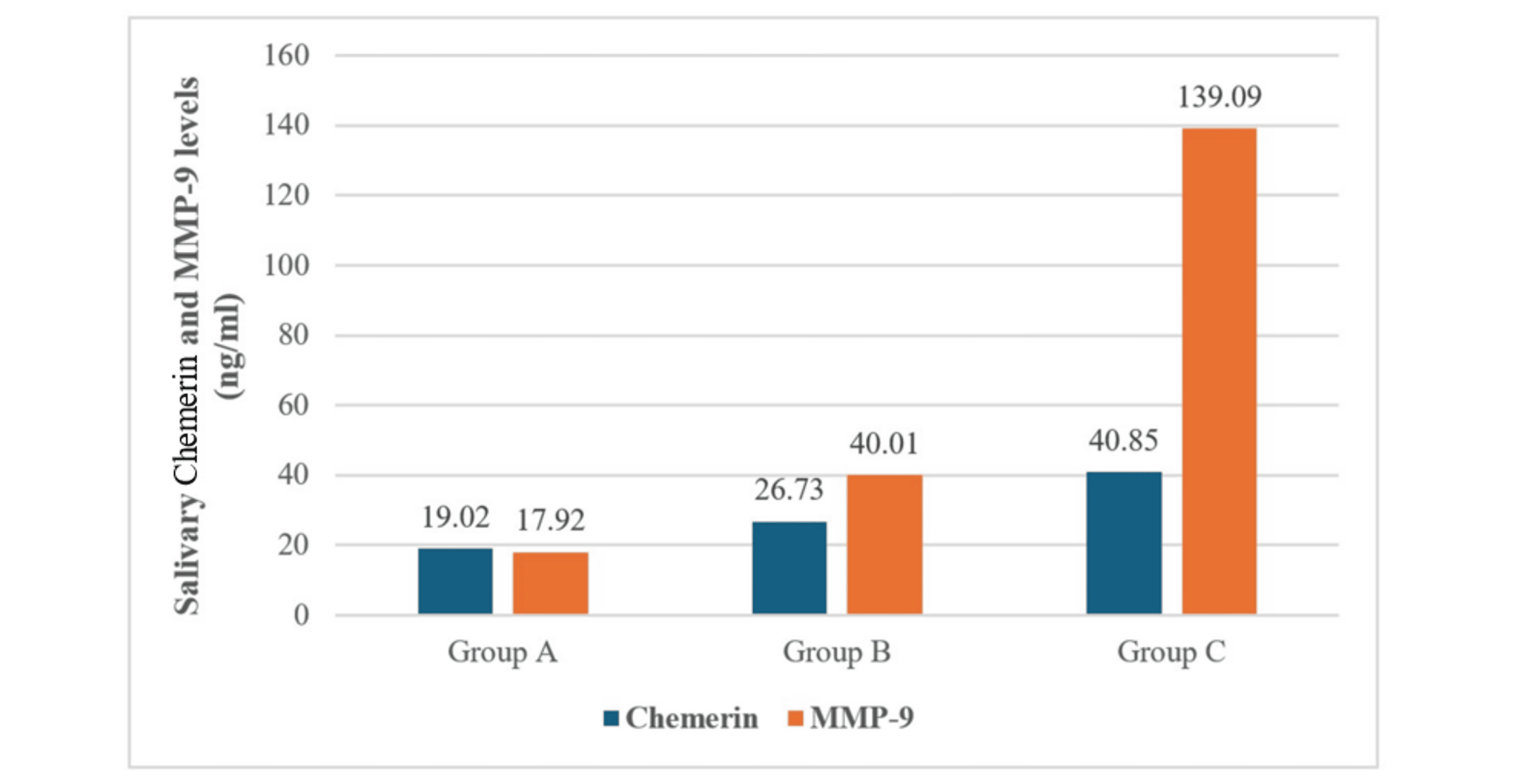
Mean salivary chemerin and MMP-9 concentration in Groups A (healthy), B (periodontitis patients), and C (OSCC patients). MMP-9: matrix metalloproteinase-9; OSCC: oral squamous cell carcinoma

Clinical assessment revealed significantly poorer periodontal status in the periodontitis (Group B) and OSCC (Group C) groups compared to healthy individuals (Group A). The mean OHI-S scores were 0.62 ± 0.25, 3.85 ± 0.52, and 2.68 ± 0.48 for Groups A, B, and C, respectively, indicating significant differences among the groups (F(2, 87) = 373.35, p < 0.001). Similarly, the mean Russell’s index scores were 0.18 ± 0.07, 3.72 ± 0.39, and 2.96 ± 0.66, respectively, with a highly significant intergroup variation (F(2, 87) = 556.28, p < 0.001). The mean PPD was 0 mm in Group A, 7.85 ± 0.92 mm in Group B, and 6.72 ± 1.05 mm in Group C. Likewise, the mean CAL was 0 mm, 4.25 ± 0.95 mm, and 3.95 ± 1.28 mm in Groups A, B, and C, respectively. Both parameters demonstrated statistically significant differences among the groups (PPD: F(2, 87) = 467.13, p < 0.001; CAL: F(2, 87) = 162.81, p < 0.001). Analysis of salivary chemerin and MMP-9 levels showed progressive increases from healthy individuals to periodontitis and OSCC. The mean salivary chemerin concentrations were 19.02 ± 3.80 ng/mL in Group A, 26.73 ± 5.18 ng/mL in Group B, and 40.85 ± 8.73 ng/mL in Group C, with a significant intergroup difference (F(2, 87) = 93.88, p < 0.001). A similar trend was observed for salivary MMP-9 levels, which increased from 17.92 ± 6.01 ng/mL in healthy individuals to 40.01 ± 18.80 ng/mL in periodontitis patients and 139.09 ± 55.22 ng/mL in OSCC patients (F(2, 87) = 109.00, p < 0.001). Overall, clinical and biomarker parameters differed significantly among groups, with the greatest deterioration and highest chemerin and MMP-9 in the OSCC group (Table 1; Figure 2). The intergroup comparison demonstrated statistically significant differences among all three groups (p = 0.000) (Table 1).

**Table 1:** An intergroup comparison of the OHI-S, Russell’s index, PPD, CAL, chemerin, and MMP-9 levels was conducted using ANOVA. *p < 0.05 was considered statistically significant. OHI-S: oral hygiene index-simplified; PPD: probing pocket depth; CAL: clinical attachment level; MMP-9: matrix metalloproteinase-9; ANOVA: analysis of variance

Parameters	Groups	Mean	Standard deviation	Standard error	95% confidence interval for mean		Minimum	Maximum	F-value	df	p-value
					Lower bound	Upper bound					
OHI-S	Group A	0.62	0.25	0.045	0.53	0.71	0.2	1.0	373.35	2, 87	<0.001*
	Group B	3.85	0.52	0.093	3.66	4.04	3.0	5.5			
	Group C	2.68	0.48	0.086	2.50	2.86	1.7	3.5			
Russell’s index	Group A	0.18	0.07	0.013	0.15	0.21	0.0	0.3	556.28	2, 87	<0.001*
	Group B	3.72	0.39	0.070	3.57	3.86	2.9	4.5			
	Group C	2.96	0.66	0.118	2.72	3.20	1.9	4.2			
PPD	Group A	0.00	0.000	0.000	0.00	0.00	0	0	467.13	2, 87	<0.001*
	Group B	7.85	0.92	0.165	7.51	8.19	6	10			
	Group C	6.72	1.05	0.188	6.34	7.10	5	9			
CAL	Group A	0.00	0.000	0.000	0.00	0.00	0	0	162.81	2, 87	<0.001*
	Group B	4.25	0.95	0.170	3.90	4.60	3	7			
	Group C	3.95	1.28	0.230	3.48	4.42	0	7			
Chemerin (ng/mL)	Group A	19.02	3.80	0.69	17.60	20.44	11.9	24.5	93.88	2, 87	<0.001*
	Group B	26.73	5.18	0.95	24.79	28.67	16.6	36.8			
	Group C	40.85	8.73	1.59	37.59	44.11	25.7	60.3			
MMP-9 (ng/mL)	Group A	17.92	6.01	1.10	15.68	20.17	9.8	30.1	109.00	2, 87	<0.001*
	Group B	40.01	18.80	3.43	32.99	47.03	6.0	75.4			
	Group C	139.09	55.22	10.08	118.47	159.71	18.5	240.5			

Tukey’s post hoc analysis revealed significant differences among the study groups for most clinical and biomarker parameters. OHI-S, Russell’s index, and PPD showed significant differences between all pairwise group comparisons (p < 0.05). For CAL, significant differences were observed between healthy individuals (Group A) and both periodontitis (Group B) and OSCC (Group C) patients (p < 0.001), whereas the difference between Groups B and C was not significant (p = 0.693). Salivary chemerin and MMP-9 levels were significantly higher in Groups B and C compared with Group A and were also significantly higher in Group C than in Group B (p < 0.001), indicating elevated biomarker expression in OSCC patients (Table 2).

**Table 2:** Intergroup comparison of the OHI-S, Russell’s index, PPD, CAL, chemerin, and MMP-9 levels using Tukey’s post hoc test. *p < 0.05 was considered statistically significant. OHI-S: oral hygiene index-simplified; PPD: probing pocket depth; CAL: clinical attachment level; MMP-9: matrix metalloproteinase-9

Dependent variable	Comparison	Mean difference (I-J)	Standard error	p-value	95% confidence interval lower bound	95% confidence interval upper bound
OHI-S	Group A vs. Group B	-3.23	0.10	<0.001*	-3.55	-2.91
	Group A vs. Group C	-2.06	0.10	<0.001*	-2.38	-1.74
	Group B vs. Group C	1.18	0.08	<0.001*	0.96	1.56
Russell’s index	Group A vs. Group B	-3.54	0.08	<0.001*	-3.58	-3.06
	Group A vs. Group C	-2.78	0.08	<0.001*	-3.13	-2.61
	Group B vs. Group C	0.76	0.08	<0.001*	0.19	0.71
PPD	Group A vs. Group B	-7.85	0.19	<0.001*	-8.22	-6.91
	Group A vs. Group C	-6.72	0.19	<0.001*	-7.55	-6.25
	Group B vs. Group C	1.13	0.19	0.045*	0.01	1.32
CAL	Group A vs. Group B	-4.25	0.20	<0.001*	-5.25	-3.89
	Group A vs. Group C	-3.95	0.20	<0.001*	-5.01	-3.65
	Group B vs. Group C	0.30	0.20	0.693	-0.45	0.91
Chemerin (ng/mL)	Group A vs. Group B	-7.71	1.32	<0.001*	-10.89	-4.53
	Group A vs. Group C	-21.83	1.32	<0.001*	-25.01	-18.65
	Group B vs. Group C	-14.12	1.32	<0.001*	-17.30	-10.94
MMP-9 (ng/mL)	Group A vs. Group B	-22.08	7.64	0.035*	-40.48	-3.68
	Group A vs. Group C	-121.17	7.64	<0.001*	-139.57	-102.77
	Group B vs. Group C	-99.09	7.64	<0.001*	-117.49	-80.69

Pearson’s correlation analysis was conducted to assess the relationship between periodontal parameters and salivary biomarker levels. The correlation coefficients showed a statistically significant result at the 0.01 level. OHI-S, Russell’s index, PPD, and CAL demonstrated strong positive correlations with salivary chemerin and MMP-9 levels. The results suggest that greater periodontal destruction and worsening clinical periodontal status are associated with elevated expression of inflammatory biomarkers. Elevated concentrations of chemerin and MMP-9 were observed in individuals exhibiting more severe periodontal involvement, suggesting their possible role in disease progression and tissue destruction (Table 3).

**Table 3:** Pearson’s correlation among OHI-S, Russell’s index, PPD, CAL, chemerin, and MMP-9 levels. **p < 0.001, indicating a highly statistically significant. OHI-S: oral hygiene index-simplified; PPD: probing pocket depth; CAL: clinical attachment level; MMP-9: matrix metalloproteinase-9

		OHI-S	Russell’s index	PPD	CAL	Chemerin	MMP-9
OHI-S	Pearson correlation	1	0.957**	0.948**	0.904**	0.801**	0.866**
	p-value	NA	<0.001	<0.001	<0.001	<0.001	<0.001
Russell’s index	Pearson correlation	0.957**	1	0.979**	0.944**	0.842**	0.899**
	p-value	<0.001	NA	<0.001	<0.001	<0.001	<0.001
PPD	Pearson correlation	0.948**	0.979**	1	0.953**	0.861**	0.913**
	p-value	<0.001	<0.001	NA	<0.001	<0.001	<0.001
CAL	Pearson correlation	0.904**	0.944**	0.953**	1	0.826**	0.879**
	p-value	<0.001	<0.001	<0.001	NA	<0.001	<0.001
Chemerin	Pearson correlation	0.801**	0.842**	0.861**	0.826**	1	0.765**
	p-value	<0.001	<0.001	<0.001	<0.001	NA	<0.001
MMP-9	Pearson correlation	0.866**	0.899**	0.913**	0.879**	0.765**	1
	p-value	<0.001	<0.001	<0.001	<0.001	<0.001	NA

## Discussion

Periodontitis is a chronic inflammatory disease caused by the host's immune response to pathogenic microorganisms, leading to progressive destruction of the periodontal tissues. This immune response activates a complex inflammatory cascade that involves mediators such as cytokines, leukotrienes, and prostaglandins, which contribute to connective tissue breakdown and alveolar bone loss [18]. Recently, salivary biomarkers such as chemerin and MMP-9 have attracted considerable attention for their roles in the pathogenesis of inflammatory diseases. Chemerin, a pro-inflammatory adipokine, regulates immune cell recruitment and inflammatory responses. MMP-9, a member of the matrix metalloproteinase family, is a key enzyme involved in the degradation of the extracellular matrix and the destruction of connective tissue [19,20]. Elevated levels of these biomarkers have been associated with increased severity of periodontal inflammation. Furthermore, both chemerin and MMP-9 have been implicated in OSCC, where chronic inflammation and oxidative stress contribute significantly to carcinogenesis [21]. Their increased salivary levels in OSCC patients suggest potential as dual-purpose biomarkers for distinguishing between inflammatory and malignant oral conditions. Given its non-invasive nature and molecular richness, saliva serves as a valuable diagnostic medium, enhancing early detection, disease monitoring, and treatment of periodontal disease and OSCC [22].

Our findings show increased periodontal indices and elevated oxidative and inflammatory biomarkers in subjects with periodontitis and OSCC, aligning with existing literature that links poor oral hygiene to oral diseases. Bakdash identified inadequate oral hygiene as a major risk factor for periodontal disease, and Moreno-López et al. found it to be an independent risk factor for OSCC [23,24].

Lertpimonchai et al. noted that fair to poor oral hygiene increases the risk of periodontitis by two to five times, aligning with our results [25]. Additionally, Farquhar et al. suggested that oral health influences tumor biology through inflammation, a finding supported by our findings of elevated biomarkers in OSCC patients [26]. Mathur et al. emphasized that poor oral hygiene worsens carcinogenic effects, suggesting that compromised periodontal health may promote OSCC through inflammatory and oxidative pathways [27].

The present study demonstrated a gradual increase in salivary chemerin levels from healthy individuals to periodontitis and OSCC patients, with the highest levels observed in the OSCC group. Statistical analysis revealed significant intergroup differences, suggesting a strong association between chemerin expression and disease severity. The elevated salivary chemerin levels found in periodontitis patients in this study align with previous research by Özcan et al., who reported elevated chemerin concentrations in inflammatory periodontal diseases and emphasized its association with periodontal tissue destruction. Similar findings have also suggested the potential role of chemerin as an inflammatory biomarker in periodontal disease [28].

The markedly elevated salivary chemerin levels identified in the OSCC group in the study are consistent with previous studies by Susha and Ravindran and other researchers who reported increased chemerin expression in OSCC and highlighted its association with tumor-related inflammation, immune modulation, and tissue destruction [29,30]. Previous studies suggest chemerin may serve as a diagnostic biomarker for early OSCC detection. In agreement with these findings, the present study demonstrated the highest chemerin levels in OSCC patients, supporting its potential role in tumor progression and its value as a non-invasive salivary biomarker for oral malignancy.

The elevated salivary MMP-9 levels identified in periodontitis patients in this study highlight its important role in periodontal inflammation and connective tissue destruction. Kim et al. also reported significantly higher salivary MMP-9 levels in patients with periodontitis compared to healthy individuals [31].

This study demonstrated significantly higher salivary MMP-9 levels in OSCC patients, supporting its role in tumor progression and tissue destruction. Comparable results have been documented by Pazhani et al., who observed progressively higher salivary MMP-9 levels from healthy individuals to oral leukoplakia and OSCC patients, with the highest levels observed in poorly differentiated tumors, indicating an association with tumor aggressiveness and disease severity [32]. Previous research has emphasized the significance of MMP-9 in extracellular matrix degradation, tumor invasion, and disease progression. In agreement with these observations, the present study demonstrated the highest salivary MMP-9 levels in the OSCC group, further supporting its potential as a non-invasive biomarker for oral malignancy.

Although salivary chemerin and MMP-9 levels were highest in the OSCC group, the periodontal clinical parameters (OHI-S, Russell’s index, PPD, and CAL) were more severe in the periodontitis group. This suggests that the elevated biomarker levels observed in OSCC may not be solely attributable to periodontal destruction. Chemerin and MMP-9 are involved in tumor-associated inflammation, extracellular matrix degradation, angiogenesis, and tumor invasion, which may contribute to their increased expression in OSCC patients. Therefore, the higher biomarker levels in the OSCC group likely reflect both cancer-related biological activity and underlying inflammatory processes rather than a simple progression of periodontal disease severity.

Pearson’s correlation analysis demonstrated strong positive associations between periodontal clinical parameters and salivary chemerin and MMP-9 levels. However, despite the periodontitis group exhibiting higher OHI-S, Russell’s index, PPD, and CAL values than the OSCC group, the highest biomarker concentrations were observed in OSCC patients. This finding suggests that factors beyond periodontal destruction may contribute to elevated chemerin and MMP-9 levels.

However, the study is limited by a relatively small sample size, which may reduce the generalizability of the findings to a broader population. Salivary biomarker levels were not evaluated and compared across different TNM stages and histopathological grades of OSCC or varying severities of periodontal disease. The study focused on a specific demographic group, which may have influenced the observed biomarker levels. In addition, oral microbial profiles were not assessed; therefore, the potential role of microbial dysbiosis in the association between periodontitis and OSCC could not be directly investigated. Further studies with detailed disease stratification and microbiological assessment are warranted to validate and expand upon the present findings.

## Conclusions

Salivary chemerin and MMP-9 levels were significantly higher in patients with periodontitis and OSCC than in healthy individuals, with the highest concentrations observed in the OSCC group. Elevated biomarker levels were associated with poorer periodontal status and may reflect underlying inflammatory and tissue-remodeling processes common to both conditions. The findings support an association between periodontitis, OSCC, and increased salivary levels of chemerin and MMP-9. However, owing to the study's cross-sectional design, no conclusions can be drawn regarding causality or diagnostic accuracy. Future large-scale longitudinal studies are needed to clarify the biological relationship between periodontitis and OSCC and to evaluate the diagnostic performance and clinical utility of these biomarkers through analyses such as sensitivity, specificity, cut-off values, and receiver operating characteristic (ROC) curves.
